# Pyrimidine Triones as Potential Activators of p53 Mutants

**DOI:** 10.3390/biom14080967

**Published:** 2024-08-08

**Authors:** Maryam M. Jebril Fallatah, Özlem Demir, Fiona Law, Linda Lauinger, Roberta Baronio, Linda Hall, Elodie Bournique, Ambuj Srivastava, Landon Tyler Metzen, Zane Norman, Rémi Buisson, Rommie E. Amaro, Peter Kaiser

**Affiliations:** 1 Department of Biological Chemistry, University of California Irvine, Irvine, CA 92697, USA; 2 Department of Chemistry and Biochemistry, University of California San Diego, La Jolla, CA 92093, USA

**Keywords:** mutant p53, tumor suppressor, CETSA, DSF, p53 corrector, p53 reactivator, cancer

## Abstract

p53 is a crucial tumor suppressor in vertebrates that is frequently mutated in human cancers. Most mutations are missense mutations that render p53 inactive in suppressing tumor initiation and progression. Developing small-molecule drugs to convert mutant p53 into an active, wild-type-like conformation is a significant focus for personalized cancer therapy. Prior research indicates that reactivating p53 suppresses cancer cell proliferation and tumor growth in animal models. Early clinical evidence with a compound selectively targeting p53 mutants with substitutions of tyrosine 220 suggests potential therapeutic benefits of reactivating p53 in patients. This study identifies and examines the UCI-1001 compound series as a potential corrector for several p53 mutations. The findings indicate that UCI-1001 treatment in p53 mutant cancer cell lines inhibits growth and reinstates wild-type p53 activities, including DNA binding, target gene activation, and induction of cell death. Cellular thermal shift assays, conformation-specific immunofluorescence staining, and differential scanning fluorometry suggest that UCI-1001 interacts with and alters the conformation of mutant p53 in cancer cells. These initial results identify pyrimidine trione derivatives of the UCI-1001 series as candidates for p53 corrector drug development.

## 1. Introduction

The p53 protein, encoded by the *TP53* gene, serves as a vital tumor suppressor in vertebrates, playing a crucial role in preventing the initiation and progression of cancer. However, most tumors have developed strategies to counteract the anti-cancer functions of p53, making the p53 pathway an attractive target for pharmaceutical cancer therapies. p53, which is present in both the nucleus and cytoplasm, acts as a transcription factor, selectively binding to DNA and governing the expression of various genes [1,2]. Under regular circumstances, the levels of cellular p53 protein remain minimal due to stringent regulation by its inhibitors Mdm2 and MdmX. These inhibitors facilitate the degradation of p53 via ubiquitylation [3,4,5,6,7]. When cells face cellular stress, such as DNA damage, nutrient deprivation, etc., the process of p53 degradation by Mdm2 is hindered. Consequently, this leads to a rapid elevation in the levels of p53 protein within the cell. p53 then prevents cell proliferation of damaged or stressed cells by triggering the transcriptional activation of different genes to induce cell cycle arrest and, eventually, cell death [8].

A relatively small subset of tumors achieves p53 suppression via enhanced degradation, attributed to Mdm2 amplification, or E6 viral ubiquitin ligase expression in human papilloma virus-induced cancers [9,10]. Meanwhile, around 40% of human cancers possess predominantly missense mutations in the *TP53* gene, leading to its diminished functionality as a tumor suppressor [11,12]. Most p53 missense mutations found in human cancers are located in the DNA binding domain, specifically at six hotspot residues (R175, G245, R248, R249, R273, R282). p53 hotspot mutations are classified into conformational mutants (R175, G245, R249, and R282) causing p53 folding instability, and DNA contact mutants (R248, R273) affecting nucleotide interaction at p53 DNA binding sites [13]. Tumors with p53 hotspot mutations are more aggressive than those lacking p53, suggesting that certain p53 mutants exhibit gain-of-function (GOF) oncogenic properties [14,15,16]. GOF activities differ among p53 mutants [17]. Missense mutations often lead to a defective yet abundant p53 protein because they escape the Mdm2-mediated degradation pathway and can therefore activate potent off-target GOF pathways [18]. Various mechanisms have been proposed to explain how GOF p53 mutants advance tumor growth. They include disabling related proteins such as p63 and p73 and interaction with DNA-binding or chromatin-remodeling factors [15]. Recent research has identified potential drugs to restore p53 tumor suppressor function in cells with mutated p53. Progress in targeting p53 missense mutations has been slow due to the challenges associated with corrector drug approaches, but recent advancements have reached clinical trials [11,17]. Compounds are classified according to their binding mode to the p53 protein, falling into either covalent or noncovalent categories. Covalently binding compounds include arsenic trioxide (ATO), an FDA-approved cysteine reactive compound that was recently shown to covalently attach to cysteines in highly unstable p53 mutants and thereby increasing folding stability [19]. APR-246, a phase 3 trial drug evolved from PRIMA-1 targets several cysteines in p53 [20,21]. Notably, the efficacy of APR-246 is more influenced by the expression of the cystine transporter SLC7A11 and cellular glutathione levels than by *TP53* mutation status, suggesting a mode of action in vivo that may not be related to p53 but influenced by induction of redox imbalance through APR-246 [22,23]. On the other hand, noncovalently binding compounds have initially focused on a cryptic binding pocket created by mutations at Y220. These compounds are selective for such p53 mutants and were pioneered by a series of PhiKan molecules [24]. More recently, the clinical compound PC14586, which also targets the specific mutation-induced cleft in p53-Y220 mutants, was reported to efficiently restore tumor suppressor activity in cells, mice, and human tumors. Early clinical results with PC14586 suggest efficacy in human cancer and establish mutant p53 reactivation as a clinically relevant therapeutic path [25]. Potassium antimony tartrate (PAT), an anti-parasitic drug, was shown to restore the thermal stability of a group of mildly unstable, temperature-sensitive p53 mutants. Antimony targets the same p53 cysteine triad as arsenic but interacts noncovalently and is thus less effective at providing structural stability [26]. Thiosemicarbazones, including ZMC1 (NSC319726) and COTI-2, are metal-chelating compounds that have shown selective killing of cancer cells carrying p53 mutations [27,28]. Delivery of zinc to stabilize p53 folding as well as generation of reactive oxygen species that deplete glutathione have been suggested as possible mechanisms [28,29]. The UCI-LC0023 compound series, comprising benzimidazole derivatives, was suggested to operate through a distinct mechanism by noncovalently binding to the L1/S3 (loop 1, sheet 3) pocket of p53 [30]. This interaction stabilizes the protein’s wild-type structure and can reactivate DNA-binding functionality in p53 mutants and impair tumor progression in a p53 mutant-dependent manner in mouse models [30]. 

In this study, we apply a computational screening approach using a molecular dynamic simulated p53 conformation. We describe identification of pyrimidine trione derivatives as potential mutant p53 correctors. The identified compound induces thermal stability of destabilized p53 mutants in vivo and in vitro, activates p53-dependent transcription and induces apoptosis specifically in p53 mutant-expressing cells.

## 2. Materials and Methods

### 2.1. Computational Screening

As the L1/S3 pocket was closed in all available crystal structures, we used the open L1/S3 pocket conformation generated by molecular dynamics simulations of the p53-R273H mutant (cluster13 representative in [31]) for a virtual screen of ChemBridge Express Pick compound library that consists of 140,000 compounds. The compounds were first filtered in the Schrodinger suite to strictly obey Lipinski’s Rule of Five and Jorgensen’s Rule of Three, as well as logPo/w < 3 (to provide better solubility). The number of reactive functional groups in compounds was enforced to be less than or equal to 3. We then used different methods to select 3 sets of compounds. Autodock Vina (version 1.1.1) [32] was used to run the virtual screen targeting a 20 × 20 × 20 Å3 search box centered on a Cys124 sulfur atom with exhaustiveness = 8. The best docking score for the entire library was −8.4 kcal/mol. Compounds that had highest docking scores (between −8.4 kcal/mol and −7.1 kcal/mol) were selected as the first set. The docking score threshold here was arbitrarily chosen to generate about 20% of the final set of compounds. For the second set, we clustered compounds with docking scores between −7.1 kcal/mol and −6.5 kcal/mol using K-means clustering of their dendritic binary fingerprints in Schrodinger’s Canvas module and selected compounds which were cluster centroids. As the third and final set, we selected the highest-scoring compounds from a TanimotoCombo similarity screen of stictic acid, the p53-reactivating candidate identified in Wassman et al. [31], using Openeye’s ROCS module. A final filter for lead-like properties in the Openeye suite left us with 87, 275, and 85 compounds for the 3 sets described, respectively. Altogether, ~450 compounds from the ChemBridge Express Pick library were selected for experimental testing. The entire selection process is summarized in Scheme (Figure S1A) in the Supplementary Information.

### 2.2. System Setup for MD Simulations

MD simulations were performed using the Desmond program available in the Schrödinger Suite. The selected binding poses of UCI-1001 and the p53 protein were subjected to one 500 ns simulation. A cubic box periodic boundary was employed, using the TIP3P water model. The simulations maintained a salt concentration of 150 mM, a temperature of 300 K, and a pressure of 1 atm. The simulation data analysis was conducted using the Simulation Interaction Diagram wizard within the Schrödinger Suite.

### 2.3. Compounds, Cell Culture, Viral Packaging, and Transfection 

**Compounds:** UCI-1001 (Cat# F0862-0015, Life Chemicals, Niagara-on-the-Lake, Canada), UCI-1014 (Cat# F0350-0188, Life Chemicals, Niagara-on-the-Lake, Canada), and APR-246 (Eprenetapopt, Cat# S7724, Selleckchem, Houston, TX, USA) were dissolved in DMSO (Cat# 67-68-5, MilliporeSigma, Burlington, MA, USA) to achieve a final concentration of 50 mM. UCI-1001 analogs were purchased from ChemBridge (San Diego, CA, USA) and MCULE (Palo Alto, CA, USA); their structures and IDs are provided in Table 1 and Table S1. Compound structures were drawn using ChemDraw 22.0. N-acetyl-cysteine (NAC) (Sigma-Aldrich, Inc., St. Louis, MO, USA) was prepared in aqueous solutions. A 1 mg/mL ATO stock solution was prepared by dissolving ATO powder in 1 M NaOH and subsequently adjusting the pH to 7.2 using HCl.

**Cell lines:** The following cell lines were obtained from ATCC: Saos-2 (osteosarcoma, female) (p53 Null), TOV-112D (adenocarcinoma, ovary, female) (p53-R175H), and MCF7 (adenocarcinoma, breast, female) (p53 wild-type). The following two cell lines were obtained from Dr. Rémi Buisson: HCT116 (colorectal carcinoma, male) (p53 wild-type) and HEK-293T (immortalized human embryonic kidney) (p53 wild-type). All cells were cultured at 37 °C in a 5% CO_2_ environment. The TOV-112D cells were maintained in a 1:1 mixture of MCDB 105 (Cat# 117-500, Sigma-Aldrich, Inc., St. Louis, MO, USA) and Gibco 199 (Cat# 12340-030, Gibco, Waltham, MA, USA) medium. Saos-2, MCF-7, HCT116 and HEK-293T cells were cultured in DMEM (Cat# 10-017-CM, Corning, Glendale, AZ, USA). All cell line media were supplemented with 10% fetal bovine serum (FBS) (Gibco, Waltham, MA, USA) and 1× Corning Antibiotic–Antimycotic Solution (Cat# 30-004-CI, Thermo Fisher Scientific, Waltham, MA, USA). Stable Saos-2 cell lines containing doxycycline-inducible (DOX) wild-type or mutant p53 were developed by a lentiviral strategy, as detailed earlier by Wassman et al. [31]. TOV-112D-SLC7A11 cells were generated by infecting the cells with a lentivirus that expresses the plenti6-SLC7A11/xCT-V5 plasmid (Plasmid #170427, Addgene, Watertown, MA, USA).

**Virus generation:** 2.2 × 10^6^ HEK-293T cells were cultured in a 10 cm dish for 24 h before transfection using BioT transfection reagent (Cat# B01-01, Paramount, CA, USA), incorporating 5 µg of the lentiviral vector (plenti6-SLC7A11/xCT-V5), 5 µg of viral structural protein (pCMVdeltaR8.91), and 1 µg of the pseudotyping vector (pMD.G). Post-transfection, the medium was replaced with DMEM supplemented with 1% BSA. The virus-containing medium was collected 24 and 48 h later, followed by centrifugation and sterilization using a 0.2 µm filter.

**Lentivirus infection:** To generate TOV-112D-SLC7A11, 2 × 10^6^ per TOV-112D cells were plated in a 6-well plate. Each well was treated with DMEM, virus-containing medium, and 8 µg/mL polybrene. The TOV-112D cells were then centrifuged for 1 h at 700× *g*. Subsequently, the medium was discarded and replaced with fresh medium. Forty-eight hours post-infection, the cells were subjected to selection with 10 µg/mL blasticidin.

### 2.4. Viability Assay and IC_50_ Determination

TOV-112D, TOV-112D-SLC7A11, MCF-7, HEK-293T, and Saos-2 cell lines were seeded in 96-well plates (Cat# 655090, Greiner, Kremsmünster, Austria) at a density of 2000 cells per well and incubated overnight. To induce p53 expression in Saos-2 cells, 1 μg/mL doxycycline was added for 16 h. Twenty-four hours after seeding, compounds at various concentrations or vehicles (DMSO for UCI-1001 series and APR-246, or water for doxorubicin) were added to the cultures. These were then incubated at 37 °C in a 5% CO_2_ environment for 72 h. 

For experiments including NAC, TOV-112D cells were plated at a density of 2000 cells per well in 96-well plates. Twenty-four hours post-seeding, the cells were treated with either DMSO (as a vehicle control) or varying concentrations of UCI-1001, UCI-1014, or APR-246, each with and without 5 mM NAC. Seventy-two hours after treatment, cell viability was assessed using the CellTiter-Glo Luminescent Cell Viability Assay (Promega, Madison, WI, USA), with measurements taken on a CLARIOstar Plus microplate reader (BMG LABTECH, Ortenberg, Germany), in accordance with the manufacturer’s protocol. The data represent averages from at least three separate experiments. IC_50_ values for the compounds were calculated using GraphPad Prism version 9.0.

### 2.5. Immunoblotting and Chromatin Fractionation

**Whole-cell lysate:** TOV-112D cells were harvested and lysed using 8 M Urea lysis buffer (200 mM NaCl, 100 mM Tris-HCl, pH 7.5, 0.2% SDS). Lysates, ranging from 7 to 10 μg, were then separated by SDS-PAGE and subsequently transferred to PVDF membranes for immunodetection.

**Chromatin Fractionation** [33]: Saos-2 cells harboring different p53 mutants, along with TOV-112D cells, were treated with DMSO (vehicle), UCI-1001, UCI-1002, UCI-1014, or derivatives for either 1 or 3 h as indicated. For fractionation assays, cell pellets were collected and washed with 1x PBS. Nuclear and chromatin fractions were then prepared using the protocol described by Mendez and Stillman [33]. Fractionation buffer A consisted of 10 mM HEPES (pH 7.9), 10 mM KCl, 1.5 mM MgCl_2_, 0.34 M sucrose, 10% glycerol, and 0.1% Triton X-100. Fractionation buffer B consisted of 3 mM EDTA and 0.2 mM EGTA. Additionally, 1 mM DTT and protease inhibitors (5 mg/mL of aprotinin, 5 mg/mL of leupeptin, 0.5 mg/mL of pepstatin A, and 0.1 mM phenylmethylsulfonyl fluoride, PMSF) were included. Equivalent amounts of protein from both the chromatin-bound extract and the combined soluble cytoplasmic and nucleoplasmic extract were separated by 10–12.5% SDS-PAGE and subsequently transferred to PVDF membranes. 

**Immunoblotting:** Cell lysates were separated using 10–12.5% SDS-PAGE. The membranes were then incubated with p53 antibody (DO-1: sc-126, Santa Cruz Biotechnology, Dallas, TX, USA), GAPDH Antibody (6C5: sc-32233, Santa Cruz Biotechnology, Dallas, TX, USA), and Histone H3 Antibody (1G1: sc-517576, a marker for the chromatin fraction, Santa Cruz Biotechnology, Dallas, TX, USA) diluted in TBS 5% non-fat milk. Blots were developed using the Supersignal West Pico PLUS Chemiluminescent Substrate (Thermo Fisher Scientific, Waltham, MA, USA). Protein quantification was performed using densitometry analysis with ImageJ 1.51j8 software (doi:10.1038/nmeth.2089) [34].

### 2.6. Cellular Thermal Shift Assay (CESTA) [35,36]

Saos-2 cells expressing the p53-G245S mutant were plated and induced with doxycycline to induce the expression of p53-G245S. After 24 h, the cells were exposed to either a vehicle or 30 μM of UCI-1001 for 3 h. Subsequently, the cells were pelleted, washed with 1x PBS, divided into several tubes, and incubated at designated gradient temperatures using a thermal cycler (Bio-Rad, Hercules, CA, USA). Cells were lysed through repeated freeze–thaw cycles. Insoluble proteins were then separated by centrifugation and discarded. The soluble fraction was subjected to immunoblot analysis. The data are presented as the mean ± SEM (*n* = 3).

### 2.7. Immunofluorescence (IF) Staining

TOV-112D or HCT116 cells were treated with UCI-1001 or DMSO for three hours on coverslips pre-treated with Poly-L-lysine hydrobromide (Cat# P6282, Sigma-Aldrich, St. Louis, MO, USA) and then subjected to immunofluorescence (IF) using the protocol described in Oh et al. [37]. Cells grown on glass coverslips were fixed with paraformaldehyde (3% paraformaldehyde and 2% sucrose in 1x PBS) for 20 min, washed twice with 1x PBS, and cells were permeabilized with a permeabilization buffer (1x PBS and 0.2% Triton X-100) for 5 min. Subsequently, cells were washed twice with 1x PBS and blocked in PBS-T (1x PBS and 0.05% Tween-20) containing 2% BSA and 10% milk for 1 h. Cells were incubated with primary antibodies for 2 h: mouse clone PAb240 (Cat# OP-29, EMD Millipore, Burlington, MA, USA) to recognize mutant p53, or mouse clone PAb1620 (Cat# OP-33, EMD Millipore, Burlington, MA, USA) for p53 wild-type. Following primary antibody incubation, coverslips were washed three times with PBS-T before incubation (1 h) with donkey anti-mouse IgG secondary antibodies conjugated to Alexa Fluor 488 (Cat# A21202, Invitrogen, Waltham, MA, USA). The cells were then stained with DAPI (5 µg/mL, Cat#D9542, MilliporeSigma, Burlington, MA, USA) for 10 min, washed one time with 1x PBS, and mounted with slow-fade mounting media (Cat# S36936, Thermo Fisher Scientific, Waltham, MA, USA). Images were captured using a Leica DMi8 THUNDER microscope (Wetzlar, Germany) and the fluorescent staining intensity was quantified using CellProfiler 4.2.7 software.

### 2.8. Flow Cytometry

First, 10^5^ TOV-112D cells were seeded in 6-well plates and treated with either DMSO (vehicle), 1, 5, and 10 μM UCI-1001, or 1 μM doxorubicin (DXR). Post-treatment, the cells were harvested and stained using Annexin V, Alexa Fluor™ 647 conjugate (Cat# A23204, Thermo Fisher Scientific, Waltham, MA, USA), and propidium iodide (PI) (Cat# 25535164, Sigma-Aldrich, Inc., St. Louis, MO, USA) in Annexin-V Binding Buffer (10 mM HEPES, pH 7.5; 140 mM NaCl; 2.5 mM CaCl_2_). The stained cells were then analyzed with an ACEA NovoCyte™ Flow Cytometer (Agilent Technologies, Inc., Santa Clara, CA, USA).

### 2.9. Recombinant Protein Purification and Differential Scanning Fluorimetry (DSF) [38]

The recombinant DNA-binding domains (DBD) of p53 mutants (WT, R175H, Y220C) were purified as described in Durairaj et al. [30]. DSF was carried out following the methodology outlined in Wu et al. [38]. In the DSF process, the melting temperatures of the p53 DBDs were determined using SYPRO Orange dye (Cat# S6650, Invitrogen, Waltham, MA, USA). Real-time melt analysis was executed using a Bio-Rad CFX Connect Real-Time PCR Thermal Cycler (Bio-Rad, Hercules, CA, USA). Purified p53 DBDs were analyzed in a 96-well plate using pre-cooled HEPES buffer (20 mM HEPES, 150 mM NaCl, pH 7.5) at a final p53 protein concentration of 6.25 μM, SYPRO Orange at a 1:1000 dilution, and 30% DMSO with each well containing a total volume of 10 μL. The fluorescence signal was recorded while incrementally raising the temperature from 10 °C to 95 °C at 0.5 °C increments every 10 s, using CFX Maestro Software (Bio-Rad, Hercules, CA, USA). Data analysis for DSF was conducted using the TSA-CRAFT online tool accessed in December 2023 [39]. Only meting curves that passed the implemented quality control (“typical curves”) were considered. All measurements were performed in a minimum of 3 biological replicates.

### 2.10. Real-Time RT-PCR and RNA-Sequencing Analysis 

**Real-Time RT-PCR**: TOV-112D cells were treated for 3 h with 10 μM of UCI-1001 or DMSO. RNA was extracted from the cells using the Qiagen RNeasy kit (Qiagen, Hilden, Germany). Subsequently, cDNA was synthesized using Superscript III Reverse Transcriptase (Cat# 11904018, Invitrogen, Waltham, MA, USA). Gene expression levels were quantified using iTaq Universal SYBR Green Supermix on a Bio-Rad CFX Connect Real-Time PCR Thermal Cycler (Bio-Rad, Hercules, CA, USA).

The sequences of the primers used in qRT-PCR are as follows: p21: Forward: 5′-TGTCCGTCAGAACCCATGC-3′ reverse: 5′-AAAGTCGAAGTTCCATCGCTC-3′, NOXA: Forward: 5′-ACCAAGCCGGATTTGCGATT-3′ and reverse: 5′-ACTTGCACTTGTTCCTCGTGG-3′, GAPDH: Forward: 5′-GGAGCGAGATCCCTCCAAAT-3′, reverse: 5′-GGCTGTTGTCATACTTCTCATGG-3′ [30].

The gene expression levels were normalized to GAPDH, and the averages were displayed along with the standard deviation derived from three replicates.

### 2.11. RNA-Sequencing

RNA samples were extracted from TOV-112D cells treated with UCI-1001, UCI-1014, and DMSO using the RNeasy kit (Qiagen, Hilden, Germany). Library generation and sequencing was performed by Novogene Corporation Inc. (Sacramento, CA, USA). Sequencing reads were quantified and aligned to the human genome GRCh38 using kallisto v0.48.0 [40]. Estimated counts of transcripts from the same Ensembl gene ID were added together and the sums were converted to integers. Differential expression analysis was performed using DESeq2 v1.38.3 [41]. 

### 2.12. Generation of Volcano Blots and Venn Diagrams 

Volcano plots were generated using ggplot2 v3.4.4 [42,43]. In samples treated with either UCI-1001 or UCI-1014, genes with log fold changes greater than 1 and adjusted *p*-values less than 0.05 were considered significant. Genes with an adjusted *p*-value < 0.001 in p53 WT compared to p53 null were shown in dark blue. Venn diagrams depicting overlapping genes with *p*-values less than 0.05 were plotted using eulerr v7.0.1 [44]. 

### 2.13. Chromatin Immunoprecipitation (ChIP)

For each ChIP sample, a TOV-112D cell pellet equivalent to 0.5 × 10^7^ cells was used. Pellets were resuspended in lysis buffer (50 mM Hepes-KOH pH 7.5, 140 mM NaCl, 1 mM EDTA pH 7.5, 1% Triton-X, 0.1% sodium deoxycholate, 1 mM PMSF, 1 µg/mL pepstatin, 1 µg/mL leupeptin) and incubated for 15 min on a nutator at 4 °C. Lysates were transferred into 1.5 mL Bioruptor^®^ Microtubes with Caps for Bioruptor^®^ Pico (Diagenode, Liege, Belgium). Samples were sonicated using the Bioruptor^®^ Pico (Diagenode, Liege, Belgium) for 14 cycles (30 s on; 30 s off). Lysates were cleared by centrifugation, and 2% of each sample was taken as input. Samples were incubated overnight on a nutator with 2 µg anti-p53 Antibody (DO-1: sc-126, Santa Cruz Biotechnology, Dallas, TX, USA) along with the no-antibody control (mock). The next day, samples were incubated with 20 µL Protein A agarose/Salmon Sperm DNA (Millipore, Burlington, MA, USA) for 2 h at 4 °C on a nutator. Beads were washed twice in lysis buffer (50 mM Hepes-KOH pH 7.5, 140 mM NaCl, 1 mM EDTA pH 7.5, 1% Triton-X, 0.1% sodium deoxycholate, 1 mM PMSF, 1 µg/mL pepstatin, 1 µg/mL leupeptin) and once in wash buffer II (50 mM Hepes-KOH pH 7.5, 500 mM NaCl, 1 mM EDTA pH 7.5, 1% Triton-X, 0.1% sodium deoxycholate, 1 mM PMSF, 1 µg/mL pepstatin, 1 µg/mL leupeptin), wash buffer III (10 mM Tris-HCl pH 8.0, 250 mM LiCl, 1 mM EDTA pH 7.5, 1% NP-40, 0.5% sodium deoxycholate, 1 mM PMSF, 1 µg/mL pepstatin, 1 µg/mL leupeptin), and 1x TE pH 8.0. All washing steps were performed at 4 °C on a nutator for 5 min. DNA was eluted from beads for 20 min at 65 °C in a thermal shaker at 1000 rpm in elution buffer (50 mM Tris-HCl pH 8.0, 10 mM EDTA pH 8.0, 2% SDS). ChIP and input samples were treated with RNase A and reverse cross-linked overnight at 65 °C. The next day, ChIP and input samples were digested with 1 µg proteinase K, and subsequent DNA was precipitated with 2.5 vol. 100% ethanol (EtOH) in the presence of 2 µg Glycogen overnight at −20 °C. The next day, samples were centrifuged at maximum speed at 4 °C. DNA pellets were washed in 70% EtOH, dried, and resuspended in DNase-free water. RT-PCR was performed on a Bio-rad CFX Connect RT-PCR machine using the Bio-rad iTaq Universal SYBR Green SuperMix (Bio-Rad, Hercules, CA, USA). Sequences of primers are listed below. Enrichment was calculated and normalized to the no-antibody control (mock).

The ChIP primers sequences are as follows: p21 Forward: 5′-GTGGCTCTGATTGGCTTTCTG-3′ reverse: 5′-CTGAAAACAGGCAGCCCAAGG-3′, PUMA: Forward: 5′-GCGAGACTGTGGCCTTGTGT-3′ reverse: 5′-CGTTCCAGGGTCCACAAAGT-3′ [28,30]. Enrichment was determined using the formula 2^−(ΔCt ChIP − ΔCt Input) and then normalized against the no-antibody control (mock).

## 3. Results and Discussion

### 3.1. Identification of UCI-1001

In 2013, Wassman and colleagues utilized computational techniques to reveal a transiently accessible binding site, referred to as the L1/S3 pocket [31]. This pocket harbors multiple second-site mutations that can reactivate mutant forms of p53 [45,46,47,48]. The presence of this pocket in various p53 variants suggests the potential development of compounds capable of simultaneously targeting multiple cancer-related mutations. 

**Figure 1:**
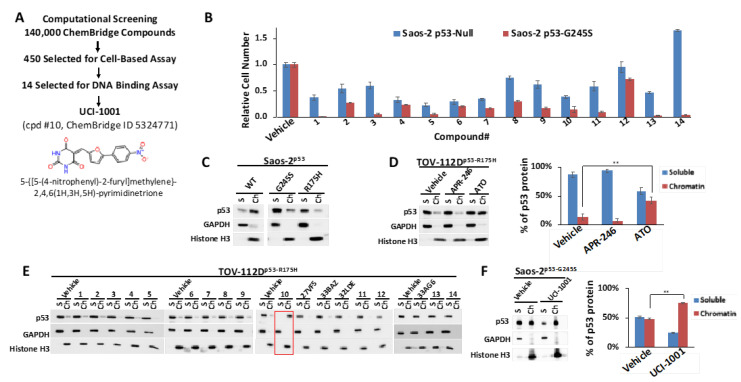
Identification of UCI-1001 (**A**) Diagram illustrating screening steps the virtual screen aimed at the L1/S3 pocket of the p53 protein resulting in identification of UCI-1001. (**B**) Saos-2 cells deficient in p53 (p53-null) or expressing mutant p53-G245S were exposed to compounds at a concentration of 10 µM (compounds **7**, **9**, **11**, and **12**) or 20 µM (compounds **1**, **2**, **3**, **4**, **5**, **6**, **8**, **10**, **13**, and **14**), for three days before the cell number was determined using the CellTiter-Glo^®^ reagent. Vehicle (DMSO) treatment served as a control. Detailed lists of the IUPAC names, structures, and IDs for the compounds can be found in Table S2. The findings are depicted as the standard error of the mean (*n* = 3). (**C**) Saos-2 cells harboring doxycycline inducible p53 (WT, G245S, or R175H) constructs were treated with 1 µg/mL of doxycycline to induce expression. Cells were fractionated into cytosolic and nuclear-soluble fractions (GAPDH as marker) and chromatin-bound fractions (histone H3 as marker). p53 distribution was detected by immunoblotting (DO-1 antibody). (**D**) TOV-112D (p53-R175H) cells were treated with 10 µM ATO or 30 µM APR-246 or DMSO (vehicle) for 24 h. Redistribution of p53 to chromatin was analyzed as in panel (**C**). The panel on the right displays quantitative analysis for 3 independent experiments. (**E**) TOV-112D (p53-R175H) cells were treated with DMSO (vehicle), 30 µM of compounds **1** to **14**, or previously published and transcriptionally inactive compounds 27VF5, 33BAZ, 32LDE, and 33AG6 [30] for 3 h. Redistribution of p53-R175H was analyzed as in panel (**C**). Red box indicates active compound #**10**. (**F**) Redistribution of p53-G245S mutants was analyzed in Saos-2 cells expressing p53-G245S; these cells were treated for 16 h with 1 µg/mL of doxycycline, followed by treatment with DMSO (vehicle) or 30 µM of UCI-1001 for 3 h. Experiment as in panel (**C**). Quantitative analysis was conducted for three independent experiments. ** indicates *p* ≤ 0.01. (Original Western Blot Images see Supplementary Materials).

To target the L1/S3 pocket, the natural compound stictic acid and benzimidazole derivatives were identified [30,31]. To extend the chemical diversity of small molecules with potential p53 mutant corrector activity, we initiated a virtual screening campaign to identify new chemical structures capable of fitting the L1/S3 pocket in p53 (Figure 1A and Figure S1A).

We assessed the ChemBridge Express Pick library, which consisted of approximately 140,000 compounds. The 450 top-ranked compounds (see Section 2.1) were selected for cell-based assays, similar to the cell-based assay screening method used to identify PRIMA-1 [49] and UCI-LC0023 [30]. We used an engineered Saos-2 cell system to experimentally validate our computationally identified compounds. Saos-2 cells lacking functional p53 were genetically modified to express wild-type (WT) p53 or p53 mutants (G245S) upon induction with doxycycline. Induction of p53-WT halted proliferation of cells, whereas induction of p53-G245S had no effect on proliferation or survival rate as compared to the parental p53-null cell [30,31]. p53-null and p53-G245S cells were exposed to the 450 selected compounds. Candidate compounds capable of reactivating p53 were distinguished from broadly cytotoxic compounds based on their differential effects on Saos-2 cells lacking p53 (p53-null) versus those expressing the p53-G245S mutant. Utilizing these specially engineered cell lines, which share an identical genetic background, enhances the reliability of the results regarding the compounds’ effects on p53 mutants, as it excludes differential compound susceptibility due to genetic variations. Out of 450 selected compounds, 14 showed selective activity in cells expressing p53-G245S (Figure 1B), compared to their effect on p53-null cells. These results helped to refine our compound selection further, ultimately focusing on 14 compounds for DNA-binding assays. Off-target effects of putative p53 mutant-reactivating compounds cannot be completely excluded by comparing effects on p53-null and p53 mutant cell lines. p53 mutant-associated gain of function properties can create vulnerabilities specifically in p53 mutant-expressing cells, which can result in differential compound sensitivity of p53-null and p53 mutant cells. One example is thiol-reactive compounds, which are more potent in some p53 mutant cell lines as compared to p53-null cells due to repression of glutathione production by some p53 mutants [22,23]. We therefore used a secondary assay, which focused on identifying compounds that can restore DNA-binding properties to p53 mutants. We utilized a chromatin fractionation protocol previously described to assess p53 activity [30,50]. We assessed the ability of compounds to promote redistribution of mutant p53 from the soluble fraction to the chromatin-bound fraction. We first evaluated chromatin fractionation as a readout for p53 activity. Saos-2 cells expressing inducible wild-type, p53-G245S, or p53-R175H were used for this assay. Cell lysates from cells expressing the different p53 versions were separated in soluble and chromatin-bound fractions. Most of the wild-type p53 is associated with chromatin in Saos-2 cells, whereas p53-R175H and p53-G245S mutants are found primarily in the soluble fraction (Figure 1C). These results agree with defects of these mutants to activate p53 target genes [51]. The chromatin fractionation assay can thus discriminate between wild-type p53 and both highly (R175H) and moderately (G245S) destabilized p53 mutants [19,38,52]. Among the six p53 hotspot mutants, p53-R175H shows the highest occurrence in cancer patients, as reported in TCGA (https://www.cancer.gov/tcga; v39.0) and the TP53 database [51]. We therefore evaluated p53 chromatin’s association with TOV-112D ovarian cancer cell lines, which express endogenous p53-R175H. We first tested the response to two well-studied compounds, APR-246 and ATO [19,53], to examine their efficacy in enabling DNA binding of endogenous p53-R175H. ATO resulted in the translocation of p53-R175H to chromatin (Figure 1D). Conversely, APR-246 did not induce a comparable redistribution of p53-R175H to chromatin at the concentration tested, confirming its limited efficacy in enabling DNA binding of p53-R175H as previously suggested [19,30]. Chromatin association tests are thus effective ways to probe DNA-binding properties of p53 and evaluate reactivation compounds in vivo. 

We used the chromatin fractionation assay to refine our selection of compounds. This assay included compounds numbered 1 through 14, along with previously reported compounds, 27VF5, 33BAZ, 32LDE, and 33AG6 [30]. Among these 14 molecules, only compound **10**, designated UCI-1001, effectively promoted redistribution of endogenous p53-R175H to the chromatin fraction in TOV-112D cells (Figure 1E). Similarly, compound **10** induced enrichment of mutant p53 on chromatin, which was observed with the moderately destabilized p53-G245S mutant ectopically expressed in Saos-2 cells (Figure 1F). Note that the moderately defective p53-G245S demonstrates partial DNA binding (48.26%), but treatment with UCI-1001 resulted in a 1.56-fold increase in the recruitment of p53-G245S to DNA, achieving a binding level of 75.18%. These results suggest that UCI-1001 has the potential to restore chromatin association of different p53 mutants to a similar extent as observed for wild-type p53. We therefore focused further characterization on UCI-1001. The chemical structure of UCI-1001 is depicted in (Figure 1A). For the chemical structures of the other compounds numbered **1**–**14**, please refer to Table S2. 

UCI-1001 induced chromatin binding of p53 mutants within 3 h. ATO required 24 h incubation with cells before reactivation could be detected in the chromatin association assay. This is consistent with timing of p53 mutant reactivation in orthogonal p53 activation assays in vivo and in vitro [19,26]. ATO acts in a covalent fashion by forming tight bonds with the cysteine triad C124, C135, and C141 in p53, which is a slow reaction and explains the delayed reactivation time. In contrast, UCI-1001 displayed a quick reactivation mode.

### 3.2. UCI-1001 Binding Is Stable 

**Figure 2:**
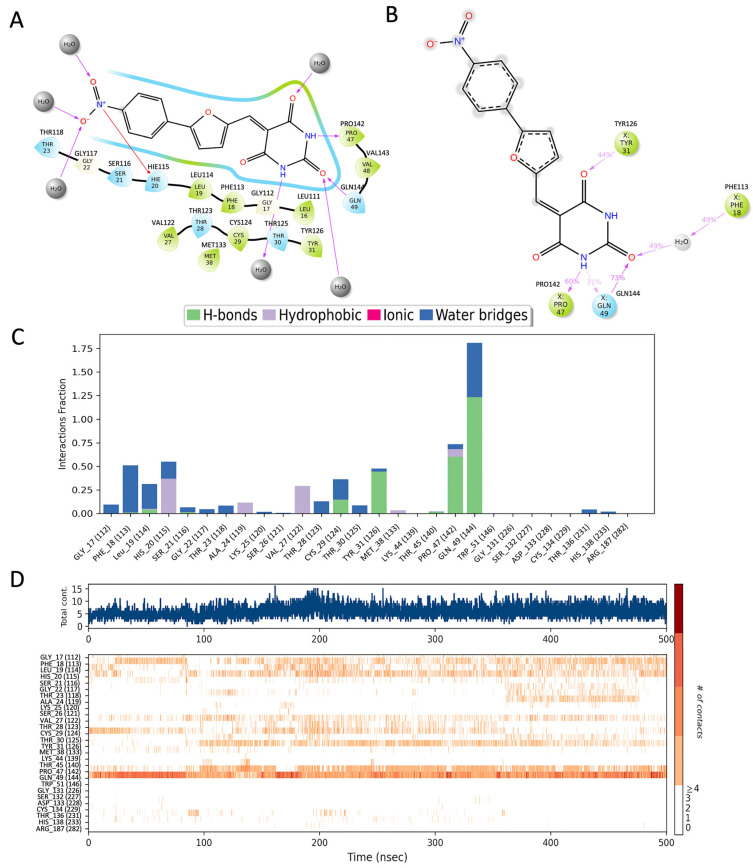
The interaction analysis of p53 with UCI-1001. (**A**) The docked structure, (**B**) residues that interact in more than 30% of the simulation trajectories, (**C**) the fraction of frames in which UCI-1001 interacts with p53 protein residues, and (**D**) the timeline of residue interactions throughout the MD simulation. Note that residues are renumbered during simulation, hence the correct residue numbers are labeled on top of (**A**,**B**) and in parenthesis in (**C**,**D**).

We explored the binding pose of UCI-1001 at the active site of p53 using the Glide docking protocol and assessed its stability through molecular dynamics (MD) simulations. To inquire binding pose of UCI-1001, we used Schrodinger’s Glide in SP mode to dock the compound to the open-L1/S3-pocket of p53 R175H (the final MD frame from 60 ns MD simulation of stictic acid bound to p53 R175H in Wassman et al. [31]). We selected one pose that is in line with SAR presented in Table 1. We then followed up with one 500 ns MD simulation using Schrodinger’s Desmond. The ligand interaction diagram reveals that the UCI-1001 compound is spatially close to hydrophilic residues such as Ser116, Thr118, Thr123, Thr125, Tyr126, and Gln144 (Figure 2A).

MD simulations indicate that the pyrimidine analog ring of UCI-1001 maintains stable interactions with Tyr126, Pro142, and Gln144, and engages in a water-mediated interaction with Phe113 (Figure 2B). The frequency of these interactions is illustrated in the frequency interaction diagram, which shows that Gln144 primarily forms hydrogen bonds, with occasional water-mediated interactions (Figure 2C). 

The timeline interaction diagram (Figure 2D) represents the number of interactions over the course of the 500 ns trajectory and the residues involved. More than five interactions between the ligand and protein are frequently observed during the simulations, with Gln144 being the most consistently interacting residue. Among the three ring structures in UCI-1001, the pyrimidine analog ring forms the most stable interactions. Future developments could focus on using the pyrimidine analog as a scaffold and modifying the rest of the molecule to achieve more specific interactions.

### 3.3. UCI-1001 Induces p53-Dependent Cell Death

**Figure 3:**
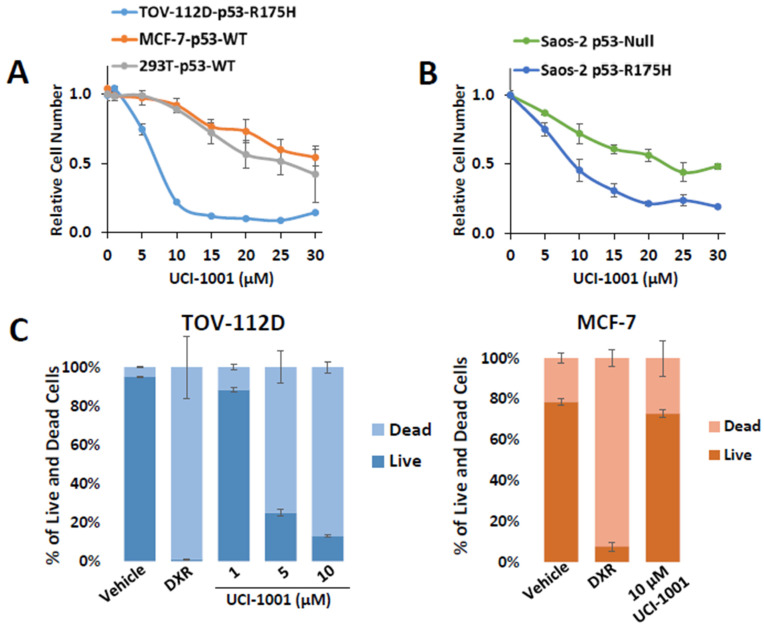
UCI-1001 inhibits cell proliferation and induces cell death in a p53 mutant-dependent manner. (**A**) Effects of different concentrations of UCI-1001 on cell proliferation/viability were evaluated for TOV-112D (p53-R175H), MCF7 (p53-WT), and HEK-293T (p53-WT) cell lines. Cell numbers were compared between compound and vehicle (DMSO) treatment after 3 days using the CellTiter-Glo^®^ reagent. (**B**) The experiment was conducted as shown in panel (**A**) but isogenic Saos-2 cells with inducible p53-R175H were compared to the parental p53-null cells. Both cell lines were cultured in the presence of doxycycline. Data are presented as the standard deviation of the mean (*n* = 3). (**C**) Cell death was analyzed by flow cytometry in TOV-112D (p53-R175H) or MCF-7 (p53-WT) cells stained with Annexin V and propidium iodide. The cells were subjected to treatment with DMSO (vehicle), 1 µM doxorubicin (DXR) as a positive control for cell death, and 1, 5, and 10 µM UCI-1001 (TOV-112D) or 10 µM for MCF-7 for 3 h. The data are presented as the standard error of the mean (*n* = 3).

We performed cell proliferation assays with TOV-112D (p53-R175H) and the two wild-type p53-expressing cell lines MCF-7 and HEK-293T. The latter expresses the SV40 large T-antigen, which binds and inactivates p53, making it functionally p53-null [54]. Cells harboring wild-type p53, p53-R175H, or lacking p53 activity responded differently to UCI-1001 in a dose–response experiment (Figure 3A). The IC_50_ for p53-R175H-expressing TOV-112D cells was 6.9 µM ± 0.1 in this experiment, whereas IC_50_ for MCF-7 and HEK-293T cells was >30 µM and 25 µM ± 1.9, respectively. Notably, cells derived from isogenic Saos-2 cell lines expressing p53-R175H (IC_50_ = 9.4 µM ± 1.9) exhibited increased sensitivity to UCI-1001 treatment compared to the parental p53-null cell line (IC_50_ = 23.9 µM ± 4.08) (Figure 3B). These results suggest that UCI-1001 exhibits selective inhibitory activity on the proliferation of cell lines harboring p53 mutations.

The tumor suppressor p53 is important for induction of cell death in cancer cells [55]. To assess whether UCI-1001 can restore the cell death-inducing activity of p53-R175H mutants, we performed Annexin V and propidium iodide staining, followed by flow cytometric analysis. Initially, we used doxorubicin (DXR) as a positive control for cell death. Both cell lines showed similar doxorubicin IC_50_ values (TOV-112D: 0.071 µM ± 0.0014, MCF-7 0.081 µM ± 0.0158) (Figure S1B) indicating similar susceptibility to cell death stimuli. We treated TOV-112D cells (p53-R175H) with 1, 5, and 10 µM of UCI-1001 for 3 h. UCI-1001 induced cell death in cells expressing mutant p53 in a dose-responsive manner (Figure 3C). Conversely, in MCF-7 cells (wild-type p53), UCI-1001 treatment resulted in minimal cell death (Figure 3C), indicating its selective activity in cancer cells harboring mutated p53.

### 3.4. UCI-1001 Induces p53 Target Genes and Promotes Association of p53-R175H with p53 Target Promoters

**Figure 4:**
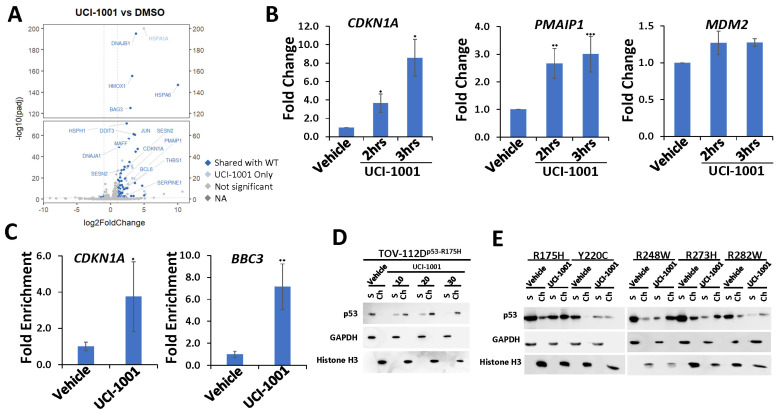
UCI-1001 induces chromatin binding and p53 target gene expression (**A**) Volcano blot depicting UCI-1001-regulated genes. Significantly changed transcripts (UCI-1001 versus DMSO) are labeled in blue. Dark blue: p53-dependent genes. Light blue: genes regulated by UCI-1001 but not by expression of wild-type p53 in Saos-2 cells. (**B**) Quantitative real-time PCR (qRT-PCR) analysis was performed to detect expression of p53 target genes *CDKN1A* (p21), *PMAIP1* (*NOXA*), and *MDM2* in TOV-112D (p53-R175H) cells treated with either 10 µM UCI-1001 or DMSO (vehicle) for 3 h. The data are presented as the standard deviation of the mean (*n* = 3). (**C**) Chromatin immunoprecipitation (ChIP) analysis reveals induction of site-specific promoter binding of p53-R175H by UCI-1001. TOV-112D (p53-R175H) cells were treated with 10 µM UCI-1001 for 30 min. The eluted DNA was analyzed by quantitative real-time PCR using primers designed to amplify the p53 binding site within the promoter regions of *CDKN1A* (p21) and *BBC3* (*PUMA*). The data are presented as the standard error of the mean (*n* = 3). (**D**) TOV-112D cells were treated with 10, 20, and 30 µM UCI-1001 or DMSO (vehicle) for 1 h, indicating dose-dependent redistribution of p53-R175H to chromatin upon compound treatment. (**E**) UCI-1001 can redistribute diverse p53 mutants to the chromatin fraction. Saos-2 cells engineered to express p53 were treated for 16 h with 1 µg/mL of doxycycline to induce expression of p53 mutants as indicated (R175H, Y220C, R248W, R273H, and R282W). Cells were treated with 25 µM UCI-1001 for 3 h. The experimental conditions were as shown in Figure 1C. * indicates *p* ≤ 0.05, ** indicates *p* ≤ 0.01 and *** indicates *p* ≤ 0.001. (Original Western Blot Images see Supplementary Materials).

The main function of p53 is activation of a specific transcription program by binding to p53-dependent promoter elements to promote gene expression [1,4,56]. p53 hotspot mutations found in human cancers are defective in transactivation of canonical p53 target genes [51,57]. The R175H mutation is structurally particularly destabilizing and p53-R175H lacks DNA binding and transactivation activity in vivo and in vitro [30,57]. We therefore evaluated genome-wide expression profiles in response to UCI-1001 exposure in the ovarian cancer cell line TOV-112D, which harbors *TP53-R175H*. As a control, we used engineered Saos-2 cell lines expressing inducible p53-WT and compared them to the parental Saos-2 (p53-null) cells to identify p53-dependent genes. TOV-112D (p53-R175H) cells were treated with 10 µM UCI-1001 for 3 h before RNA was harvested. Analyses of the expression profiles showed that 66% of UCI-1001-responsive genes in TOV-112D cells were identified as p53-dependent genes in the Saos-2 cell pair (Figure 4A). We next analyzed the expression of three canonical p53-target genes by quantitative RT-PCR two and three hours after exposure to UCI-1001. UCI-1001 rapidly upregulated the cell cycle inhibitor gene *CDKN1A* (p21) and the pro-apoptotic gene *PMAIP1* (*NOXA*). Transcription of the p53 target gene *MDM2*, a major negative regulator of p53, was not significantly induced after 3 h of UCI-1001 treatment (Figure 4B). It is possible that *MDM2* expression requires a longer exposure to the compound. Consistent with the gene expression data, UCI-1001 facilitated the re-establishment of site-specific DNA binding of the p53-R175H mutant to the promoter regions of p53-responsive genes. We evaluated binding of p53-R175H to promoters of the canonical p53-dependent cell cycle inhibitor *CDKN1A* (p21) and pro-apoptotic gene *BBC3* (*PUMA*) by chromatin immunoprecipitation (ChIP) assays in TOV-112D cells (Figure 4C). Collectively, these findings corroborate the hypothesis that UCI-1001 partially reinstates the DNA-binding capacity and transactivation activity of severely destabilized p53-R175H. These results are consistent with chromatin association assays using p53-R175H (Figure 1E) and p53-G245S (Figure 1F). Furthermore, UCI-1001 induced rapid redistribution of p53-R175H to chromatin in a dose-dependent manner showing complete chromatin association after 1 h exposure of TOV-112D cells to 30 µM of the compound (Figure 4D). We next analyzed engineered Saos-2 cell lines harboring other frequent *TP53* mutations, including structural mutants R175H, Y220C, R282W, and DNA contact mutants R248W and R273H [13]. Post-UCI-1001 treatments, although in varying degrees, examined p53 hotspot mutants transitioned from the soluble to the chromatin fraction (Figure 4E). It is notable that several of the p53 mutants showed a decreased protein concentration after compound treatment. This was particularly pronounced for p53-Y220C and R282W mutants (Figure 4E). As previously reported, p53 mutations can alter conformations so that mutant p53 is no longer targeted by the Mdm2 ubiquitin ligase and thus is highly stabilized. However, treatment with corrector compounds may restore a wild-type-like conformation that is recognized by Mdm2 leading to reduced protein levels of the reactivated p53 mutants [28,30].

Together, these results suggest that UCI-1001 may effectively restore DNA-binding functionalities across diverse p53 mutants. Restoration of chromatin association for structural mutants can be rationalized by compound-mediated stabilization of a wild-type-like conformation in p53 mutants [19,49,58]. Reactivation of DNA contact mutants is mechanistically less understood and more difficult to explain, but hotspot contact mutants also thermally destabilize p53 [59], suggesting compound-mediated stabilization of an active conformation could contribute to reactivation. Furthermore, p53 contact mutants can be rescued by small structural perturbations such as single intragenic second-site mutations [46,60,61,62].

### 3.5. UCI-1001 Promotes Conformational and Thermal Stability of p53

**Figure 5:**
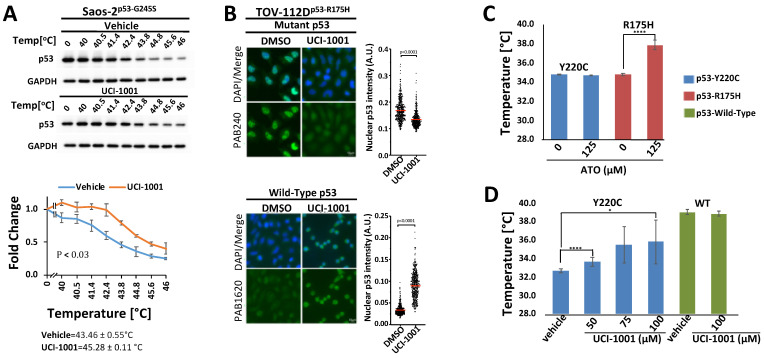
UCI-1001 promotes conformational and thermal stability of p53-R175H (**A**) Cellular thermal shift assay (CETSA) was conducted with Saos-2 cells expressing p53-G245S. The cells were treated with vehicle or 30 μM UCI-1001 for 3 h before being pelleted, distributed into several tubes, and incubated at the temperatures indicated for 3 min. Insoluble proteins were separated by centrifugation, and the soluble fraction was analyzed by immunoblotting. Data are represented as the mean ± SEM (*n* = 3). (**B**) UCI-1001 induced conformational changes in p53-R175H in TOV-112D cells, as evidenced by immunofluorescent staining with p53-conformation-selective antibodies PAB240 (mutant conformation) and PAB1620 (WT conformation). Cells in the top panel were treated with 10 μM, while those in the bottom panel were treated with 20 μM UCI-1001 for 3 h. (**C**) Differential Scanning Fluorimetry (DSF) was performed. The well-established ATO (125 µM) was analyzed as a positive control with H_2_O as vehicle comparison. ATO thermally stabilized p53-R175H but not p53-Y220C in vitro. (**D**) Various concentrations of UCI-1001, as indicated, were compared to vehicle (DMSO). The reactions contained 6.25 μM of p53-R175H, -Y220C, or -WT core domain protein. Thermal stabilization was measured using DSF. Compound incubation was 24 h for ATO and 40 min for UCI-1001. Data points are plotted as the means of three or more measurements. * Indicates *p* ≤ 0.05, and **** indicates *p* ≤ 0.0001.

Thermal stabilization and modulation of conformation are important features of p53 mutant reactivation [19,49,58]. We performed cellular thermal shift assays (CETSA) [35,63] to test UCI-1001 binding to p53 in vivo and measure the compound’s effects on thermal stability in cells (Figure 5A). We selected the less severe destabilized p53-G245S mutant for these experiments because p53-R175H tends to aggregate. Saos-2 cells expressing the conformational mutant p53-G245S were treated with UCI-1001, and cells were distributed into several aliquots and heated to different temperatures. Heat-denatured proteins were separated by centrifugation and the amount of soluble p53-G245S was determined. UCI-1001 significantly enhanced the thermal stability of p53-G245S as compared to vehicle treatment (Figure 5A). 

These findings suggest that UCI-1001 binds to p53 in vivo, leading to the thermal stabilization of p53-G245S. Moreover, the binding and modulation of p53 mutant conformation by UCI-1001 were further supported by immunofluorescence experiments using conformation-specific antibodies (Figure 5B). Specifically, these antibodies selectively identify the wild-type conformation of p53 (recognized by PAB1620) or the altered conformation of the p53-R175H mutant (recognized by PAB240 [64,65]). We focused these experiments on the most severe structural mutant, p53-R175H. When TOV-112D cells (p53-R175H) were treated with UCI-1001, there was enhanced detection using antibodies specific to the wild-type conformation, alongside a reduction in the signal specific to the p53-R175H mutant conformation. The observed decrease in the mutant conformation signal was more pronounced than the increase in the wild-type conformation signal. This observation aligns with prior studies and might be attributed to the generally lower signal intensity of the wild-type specific antibody PAB1620 [28,30]. It may also reflect the inherent characteristics of compound-mediated reactivation of p53 mutants, potentially leading to a partial restoration of the wild-type conformation. 

To determine if UCI-1001 affects detection of p53-WT with the wild-type conformation-selective antibody PAB1620, we used the HCT116 (p53-WT) cell line. Our results showed a subtle but significant increase in detection by PAB1620 after treatment with UCI-1001 (Figure S1C). This subtle increase in the p53-WT signal could suggest that UCI-1001 stabilizes an active conformation even in p53-WT. Such a scenario is feasible because p53 is thermodynamically metastable at 37 °C with a half-life for spontaneous unfolding in vivo of 10–20 min [66]. However, it is important to note that UCI-1001 did not increase the thermal stability of the recombinant DNA-binding domain p53-WT in vitro (Figure 5D), indicating that more complex events occur in vivo where p53 forms dimers and tetramers, which cannot be analyzed in our in vitro experiments. These findings confirm the results from CETSA that UCI-1001 modulates p53 mutant conformations and expand these observations to indicate a shift from a mutant conformation towards a structure akin to that of the p53 wild-type in vivo.

Both CETSA and immunofluorescence provide strong indication of compound effects on mutant p53 conformation in vivo. To further evaluate UCI-1001’s effects on p53 mutants and exclude indirect mechanisms, we used differential scanning fluorimetry (DSF) using recombinant purified p53. DSF has been previously conducted to evaluate compound effects on p53 mutants. A fluorescence dye is used to monitor unfolding of proteins in these experiments. Fluorescence emissions are systematically recorded at various temperature levels, constructing a thermal unfolding curve that aids in calculating the protein’s melting temperature, thus providing insights into its thermal stability [31,38,58,67]. To ensure the functionality of the DSF assay, we analyzed the well-characterized compound arsenic trioxide (ATO) as a positive control [19]. Incubation of ATO with the p53-R175H mutant DNA-binding domain (DBD) resulted in an increase in the medium melting temperature from 34.8 to 38.1 °C (Figure 5C). ATO did not enhance the stability of the p53-Y220C DBD (Figure 5C). This is consistent with previous reports that p53-Y220C mutations, situated distantly from the As-binding [19], were not rescued by ATO as measured using wild-type conformation-specific antibodies (PAb1620) or induction of p53-target gene expression [19,68]. In contrast to ATO, UCI-1001 is poorly soluble in water and requires DMSO as a solvent. The recombinant p53-R175H DBD is relatively sensitive to DMSO. We therefore evaluated thermal stabilization of p53 mutants by UCI-1001, using recombinant p53-Y220C, which tolerated up to 30% DMSO without dramatic protein unfolding. In contrast to ATO, UCI-1001 shows reactivation activity toward p53-Y220C (Figure 5D). Specifically, UCI-1001 significantly increased the thermal stability of the p53-Y220C DBD in a dose-dependent manner (Figure 5D). UCI-1001 did not affect the thermal stability of the DNA-binding domain of p53-WT in vitro. These results suggest that UCI-1001 directly and selectively binds to mutant p53 and can stabilize a more compact conformation. 

### 3.6. UCI-1001 Analogs

**Figure 6:**
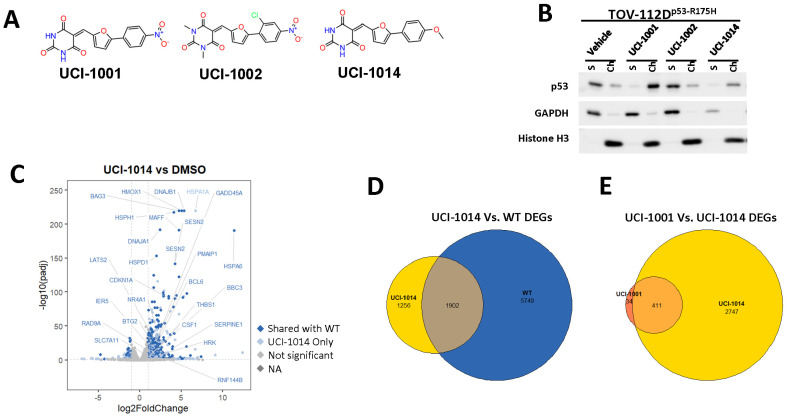
Activity of selected UCI-1001 Analogs (**A**) Chemical structures of UCI-1001, UCI-1002, and UCI-1014. (**B**) Compound activities to redistribute p53-R175H to chromatin. TOV-112D cells (p53-R175H) were treated with DMSO (vehicle), 30 µM UCI-1001, UCI-1002, or UCI-1014 for 3 h. Chromatin binding was assayed as described in Figure 1C. (**C**) Volcano blot depicting UCI-1014 regulated genes as determined by RNAseq. Significantly changed transcripts (UCI-1014 versus DMSO) are labeled in blue. Dark blue: p53-dependent genes. Light blue: genes regulated by UCI-1001 but not by expression of wild-type p53 in Saos-2 cells. (**D**) Venn diagram showing overlap between wild-type p53-dependent genes (in Saos-2 cells) and UCI-1014 controlled genes in TOV-112D. (**E**) Venn diagram showing overlap between UCI-1001 and UCI-1014 regulated genes (TOV-112D). (Original Western Blot Images see Supplementary Materials).

We acquired a collection of UCI-1001 derivatives from the repositories of ChemBridge and MCULE, each exhibiting approximately 99% structural homology with UCI-1001 while introducing subtle alterations in their molecular structures (Table 1). The key aims of these molecular modifications were to augment solubility and to evaluate structure–function relationships. To determine the efficacy of these derivatives, cell proliferation/viability assays were performed with TOV-112D cells (p53-R175H) to measure half-maximal inhibitory concentrations (IC_50_) of UCI-1001 analogs (Table 1 and Figure S2A). Several UCI-1001 derivatives showed significantly improved efficacy in cell-based assays, most notably UCI-1002, with a 10-fold reduction in the IC_50_ value (Figure S2A, Table 1). To determine whether compounds restore mutant p53 DNA-binding capacity, we conducted chromatin fractionation assays as a secondary screen for UCI-1001 analogs with improved IC_50_ values (Figure S3, Table 1). Similar to a few other compounds with enhanced potency, UCI-1002 failed to redistribute p53-R175H to chromatin, suggesting that the reduced cell proliferation/viability is independent of reactivation of mutant p53 (Figure 6A,B, Figures S3 and S4). Consistent with the results of the chromatin fractionation assay, UCI-1002 did not induce p53 target gene expression (Figure S4).

This was further supported by cell viability assays comparing TOV-112D (p53-R175H) and MCF-7 cells (wild-type p53) (Figure S2B). Both cell lines showed similar sensitivity to UCI-1002 indicating toxicity mechanisms independent of p53 status. In general, with some exceptions such as UCI-1002, compounds with lower IC_50_ did also induce redistribution of p53-R175H to chromatin, whereas higher IC_50_ values correlated with a compounds inability to restore DNA binding to p53 mutants (Figure S3 and Table 1). 

These experiments did not identify compounds with significant improvements in activity but offer valuable perspectives on the structure–activity relationships and elucidated key structural elements crucial for the specificity of effects on p53. For example, the addition of a chlorine atom to the 4-nitrophenyl moiety of UCI-1001 and substitution of hydrogen with methyl groups on nitrogen in the pyrimidine trione moiety, creating UCI-1002, significantly reduced or eliminated mutant p53 reactivation activity (Figure 6A,B and Figure S2B). In general, the introduction of methyl groups in the pyrimidine trione blocked compound effects on mutant p53 redistribution to the chromatin fraction (Figure 6A,B and Figure S3, Table 1). UCI-1001 has poor solubility even in DMSO. Interestingly, replacing the nitroxy group with a methoxy in UCI-1014 (Figure 6A) improved compound solubility in DMSO while retaining its ability to restore the DNA binding of p53-R175H mutants (Figure 6B). We therefore profiled genome-wide expression changes in response to UCI-1014 in RNAseq experiments (Figure 6C). Experiments were conducted in TOV-112D (p53-R175H) cells and expression profiles were compared between DMSO and UCI-1014 treatment for 3 h. In total, 65% of all UCI-1014 regulated genes were identified as p53-dependent genes in the isogenic Saos-2 cell system comparing p53-null with p53-WT expressing cell lines (Figure 6C,D). Interestingly, there was a very strong similarity between UCI-1001 and UCI-1014 controlled expression profiles (92% overlap) (Figure 6E). When compound-modulated genes were filtered for those that showed p53 dependence in the Saos-2 cell system, 91% of p53-dependent genes induced by UCI-1001 were also induced by UCI-1014, indicating a very similar effect of these compounds on p53 reactivation. However, the number of p53-dependent genes induced after 3 h by UCI-1014 was significantly larger as compared to UCI-1001 (3158 versus 445). This may indicate a stronger reactivation effect of UCI-1014, although the IC_50_ values of the two compounds are very similar (Table 1). It is also possible that UCI-1014 enters cells more rapidly than UCI-1001 and the relatively short exposure time of 3 h was not sufficient for expanded stimulation of target genes by UCI-1001.

### 3.7. UCI-1001 and Thiol Reactivity

**Figure 7:**
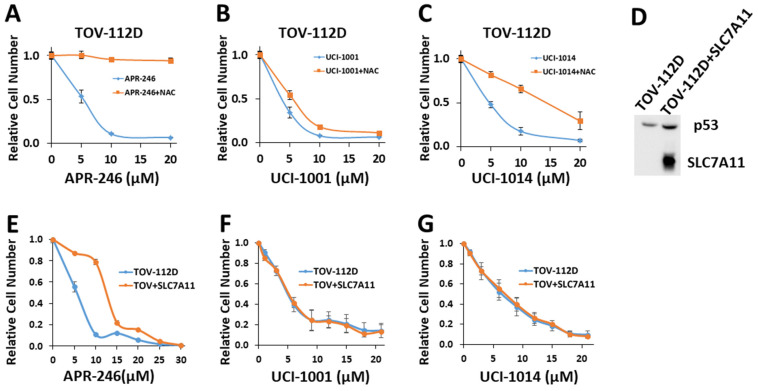
UCI-1001′s mode of action is independent of changes in redox balance (**A**–**C**) Cell viability assays were performed with TOV-112D cells (p53-R175H) treated with vehicle, 5 μM, 10 μM, and 20 μM APR-246, UCI-1001, or UCI-1014 in the presence or absence of 5 mM NAC for 3 days. Cell viability was measured using the CellTiter-Glo^®^ reagent. Data are presented as the standard deviation of the mean (*n* = 3). (**D**) Western blot of TOV-112D cells overexpressing V5-tagged SLC7A11 (**E**–**G**) Cell viability assays were conducted on TOV-112D and TOV-112D cells overexpressing SCL7A11 (referred to as TOV + SLC7A11). Cells were treated with vehicle or various concentrations of APR-246, UCI-1001, or UCI-1014 for 3 days. Cell viability was measured using the CellTiter-Glo^®^ reagent. Data are presented as the standard deviation of the mean (*n* = 3). (Original Western Blot Images see Supplementary Materials).

In the literature, a number of thiol-reactive compounds have been suggested to have mutant p53 reactivation activity [11]. The most prominent example is PRIMA-1 and its clinical derivative APR-246 [49]. While there is ample evidence that APR-246 can restore some activities of mutant p53, it is likely that the in vivo mode of action leading to induction of cell death in cancer cells expressing mutant p53 is related to the induction of redox imbalance through glutathione depletion [22,23,69]. Consequently, the suppressive effect of APR-246 on cell proliferation can be counteracted by externally administering the cell-permeable antioxidant N-acetyl-cysteine (NAC) [22]. UCI-1001 and its derivatives contain Michael acceptors and are therefore potentially cysteine-reactive. Note that APR-246 failed to redistribute p53-R175H to chromatin, whereas UCI-1001 and several derivatives as well as the well-established p53 reactivation compound ATO efficiently induced DNA binding in these assays (Figure 1D,E, Figure 4D,E, Figure 6B and Figure S3). To further test whether the observed reduction in cell proliferation/viability induced by UCI-1001 and UCI-1014 are linked to potential redox balance modification caused by Michael acceptor activity, we evaluated the effect of antioxidant (NAC) supplementation (Figure 7). TOV-112D (p53-R175H) cancer cells were subjected to these compounds in the presence of 5 mM NAC. Consistent with previous results [22,30], the introduction of NAC significantly diminished the anti-proliferative impact of APR-246 (Figure 7A). Conversely, NAC was ineffective in reversing the growth inhibition triggered by UCI-1001 (Figure 7B). NAC partially alleviated the effects of UCI-1014, but this mitigation began to diminish at a concentration of 20 μM, whereas APR-246 effects were completely quenched at this concentration (Figure 7A,C). These findings indicate that UCI-1001 and its derivatives function via a mechanism distinct from APR-246 and other thiol-reactive compounds.

To further address the issue of indirect mode of action due to induction of redox imbalance, we generated TOV-112D (p53-R175H) cell lines overexpressing the cystine transporter solute carrier family 7 member 11 (SLC7A11) (Figure 7D). SLC7A11 levels correlate with oxidative stress sensitivity in cancer cells, as it is critical to import cysteine, the rate-limiting component of glutathione synthesis [69]. An SLC7A11-mediated mechanism has been proposed to explain the selective sensitivity of cancer cells expressing p53 mutants to thiol-reactive compounds. Accumulation of mutant p53 protein inhibits SLC7A11 expression, resulting in heightened basal oxidative stress and diminished cellular ability to neutralize reactive oxygen species [22,70]. Therefore, mutated p53 effectively makes cancer cells more vulnerable to oxidative stress, as APR-246 further depletes glutathione, exacerbating the situation [22,70]. Accordingly, efficacy of APR-246 appears to be more significantly affected by the expression levels of SLC7A11 rather than by the p53 mutation status [22,23]. Consistent with this model, SLC7A11 overexpression significantly increased resistance of TOV-112D (p53-R175H) cells to APR-246 from IC_50_ = 7.3 ± 1.8 to 12.7 ± 2.8 (Figure 7E). This result aligns with a previous study [22], which demonstrated that overexpression of SLC7A11 triggered resistance to APR-246 in cancer cells with p53 mutations. Conversely, reducing SLC7A11 through knockdown heightened the susceptibility of p53-null cells to APR-246 [23]. Importantly, overexpression of SLC7A11 had no significant effect on the sensitivity of cells to UCI-1001 or UCI-1014 (Figure 7F,G). These results further support that the pharmacological activity of UCI-1001 and UCI-1014 is different from APR-246 and independent of SLC7A11 expression.

## 4. Summary and Limitations of Study

We report a computational approach to identify small molecules with the potential of reactivation of wild-type-like functions of p53 missense mutants prevalent in human cancers. We demonstrate that the pyrimidine trione derivative UCI-1001 prevents cell proliferation and induces cell death preferentially in cells expressing p53 missense mutants as compared to p53-WT harboring cells. Furthermore, we showed that UCI-1001 promotes chromatin association of various p53 missense mutants, induces thermal stabilization in vivo and in vitro, and stimulates a conformational switch of p53-R175H mutants to a wild-type-like structure in vivo. UCI-1001 triggers promoter-specific binding of p53-R175H mutants in vivo and induces expression profiles that strongly overlap with p53-WT-induced expression programs. Most of these studies were performed with cells expressing the most severe of the structural missense hotspot p53 mutants, p53-R175H. Some experiments could not be performed with p53-R175H mutants due to their strong destabilizing effect, which was further enhanced by the requirement of high concentrations of DMSO in in vitro studies to maintain UCI-1001 solubility at sufficiently high concentrations. In these cases, moderately destabilizing p53 missense mutants such as p53-G245S or p53-Y220C were used. The ability of UCI-1001 to induce chromatin association was tested in a panel of p53 missense mutants (Figure 4E). These included the two DNA contact mutants R273H and R248W. Surprisingly, UCI-1001 did induce redistribution of these mutants to chromatin. This is unexpected and mechanistically difficult to explain, although other compounds have been suggested to restore the DNA binding of p53 DNA contact mutants [30,71,72]. In addition, second-site intragenic mutations distant from the DNA contact region have been shown to compensate for the compromised DNA contact conferred by such p53 mutants [46]. The underlying mechanisms for reactivation of p53 DNA contact site mutants remain to be elucidated. We tried to exclude known indirect effects as the mechanism for UCI-1001’s mode of action. Specifically, we did not detect a significant contribution of redox imbalance to UCI-1001 activity in cell-based assays. However, lacking any structural information on p53/UCI-1001 complexes, we cannot exclude unknown indirect modes of action as explanation of p53 mutant reactivation by UCI-1001.

## Figures and Tables

**Table 1:** UCI-1001 analogs.

Name	IC_50_ (μM)	Structure	Name	IC_50_ (μM)	Structure
UCI-1001	4.1 ± 0.71	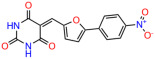	UCI-1031	21.6 ± 0.03	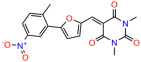
UCI-1002	0.4 ± 0.10	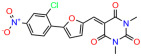	UCI-1032	23.1 ± 0.08	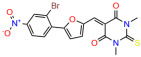
UCI-1003	1.7 ± 0.04	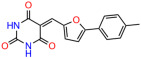	UCI-1033	14.2 ± 0.15	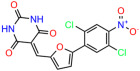
UCI-1004	1.8 ± 0.07	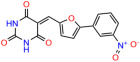	UCI-1034	9.8 ± 0.07	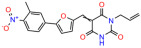
UCI-1005	2.0 ± 0.02	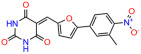	UCI-1035	11.5 ± 0.15	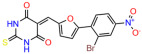
UCI-1006	2.0 ± 0.06	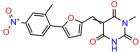	UCI-1036	12.4 ± 0.16	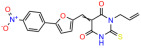
UCI-1007	2.1 ± 0.08	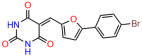	UCI-1037	22.4 ± 0.11	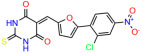
UCI-1008	2.5 ± 0.04	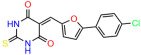	UCI-1038	15.7 ± 0.14	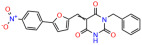
UCI-1009	3.3 ± 0.12	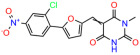	UCI-1039	14.6 ± 0.10	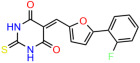
UCI-1010	3.1 ± 0.09	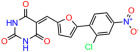	UCI-1040	14.6 ± 0.02	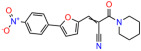
UCI-1011	4.2 ± 0.09	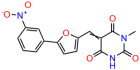	UCI-1041	15.0 ± 0.05	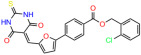
UCI-1012	3.2 ± 0.03	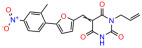	UCI-1042	17.3 ± 0.05	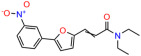
UCI-1013	4.0 ± 0.11	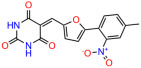	UCI-1043	16.2 ± 0.19	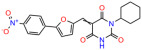
UCI-1014	4.5 ± 0.5	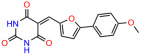	UCI-1044	16.2 ± 0.08	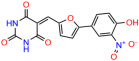
UCI-1015	3.8 ± 0.01	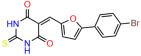	UCI-1045	16.3 ± 0.07	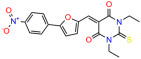
UCI-1016	3.3 ± 0.06	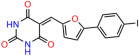	UCI-1046	15.9 ± 0.05	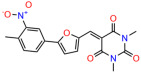
UCI-1017	3.5 ± 0.05	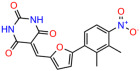	UCI-1047	17.1 ± 0.07	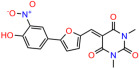
UCI-1018	4.6 ± 0.04	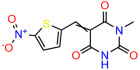	UCI-1048	16.7 ± 0.06	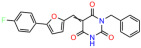
UCI-1019	4.7 ± 0.07	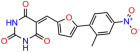	UCI-1049	16.9 ± 0.07	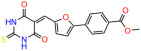
UCI-1020	5.5 ± 0.11	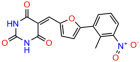	UCI-1050	>30	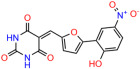
UCI-1021	5.4 ± 0.12	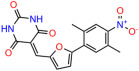	UCI-1051	25.9 ± 0.04	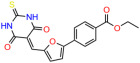
UCI-1022	6.3 ± 0.11	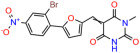	UCI-1052	27.3 ± 0.02	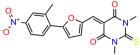
UCI-1023	6.0 ± 0.09	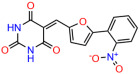	UCI-1054	3.5 ± 0.10	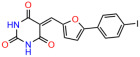
UCI-1024	6.3 ± 0.23	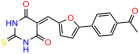	UCI-1055	4.6 ± 0.03	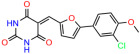
UCI-1025	6.2 ± 0.05	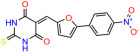	UCI-1056	6.4 ± 0.06	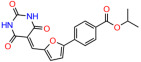
UCI-1026	6.5 ± 0.16	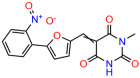	UCI-1057	6.0 ± 0.01	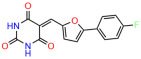
UCI-1027	6.3 ± 0.07	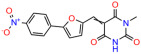	UCI-1058	6.0 ± 0.09	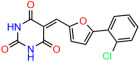
UCI-1028	7.2 ± 0.03	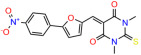	UCI-1059	2.4 ± 0.05	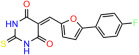
UCI-1029	6.7 ± 0.10	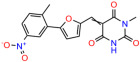	UCI-1060	4.3 ± 0.04	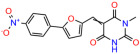
UCI-1030	6.7 ± 0.09	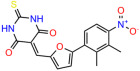	UCI-1061	4.5 ± 0.02	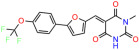

## Data Availability

RNA-sequencing data and results have been deposited in the NCBI Gene Expression Omnibus (GEO) with accession number GSE273169.

## Supplementary Materials

The following supporting information can be downloaded at: https://www.mdpi.com/article/10.3390/biom14080967/s1, Figure S1: Compound selection, doxorubicin sensitivity, and p53 conformation; Figure S2: UCI-1001 analogs IC50; Figure S3: UCI-1001 analogs chromatin binding; Figure S4: UCI-1002 does not induce p53-target gene expression; Table S1: UCI-1001 Analogs IDs; Table S2: Chemical Structures and ID for screening compounds.
