# Supplementary material for: Pyrimidine Triones as Potential Activators of p53 Mutants
Source: Biomolecules. 2024 Aug 8;14(8):967. doi: 10.3390/biom14080967 (PMC11352488; doi:10.3390/biom14080967)
Supplement: Supplementary file 1 [file biomolecules-14-00967-s001.zip › biomolecules-3012619-supplementary 8.8.pdf]

biomolecules

MDPI

Supplementary Figure Legends

Figure S1. Compound selection, doxorubicin sensitivity, and p53 conformation

A) Compound selection workflow schematic. **B)** Cell survival at different concentrations of Doxorubicin (0, 0.01, 0.10, and 1 μM) was evaluated for TOV-112D (p53R-175H) and MCF7 (p53-WT) cell lines. Cell numbers were compared between compound and vehicle (water) treatments after 3 days using the CellTiter-Glo® reagent. **C)** UCI-1001 induced a slight increase in p53 detection with PAB1620 in HCT116 cells (p53-WT) treated with 20 μM UCI-1001 for 3 hours, as evidenced by immunofluorescent staining with the p53-conformation-selective antibody PAB1620 (p53-WT conformation).

Figure S2. UCI-1001 analogs IC_{50}

A) TOV-112D cells were treated with vehicle or various concentrations of UCI-1001 analogs for 3 days. Cell viability was measured using the CellTiter-Glo® reagent. Data are presented as the standard deviation of the mean (n=3). **B)** The TOV-112D (p53-R175H) or MCF-7 (p53WT) cancer cells were treated with vehicle or various concentrations of UCI-1002 analogs for 3 days. Cell viability was measured using the CellTiter-Glo® reagent. Data are presented as the standard deviation of the mean (n=3).

Figure S3: UCI-1001 analogs chromatin binding

TOV-112D cells were treated with DMSO (vehicle) or 10μM UCI-1001 analogs for 3 hours. For chromatin binding analyses, cells were fractionated into cytosolic and nuclear soluble portions (combined), along with chromatin fractions. The chromatin fractionation was analyzed by immunoblotting using antibodies directed against p53 (DO-1), GAPDH, and Histone H3.

Figure S4: UCI-1002 does not induce p53-target gene expression

Quantitative real-time PCR (qRT-PCR) was used to measure the expression of p53 target genes *CDKN1A* (p21) and *PMAIP1* (NOXA) in TOV-112D (p53-R175H) cells treated with 1 μM UCI-1002 or DMSO (vehicle) for 3 hours. Data are presented as the standard deviation of the mean (n=3). Note that 1μM UCI-1002 was chosen based on the IC_{50} level of UCI-1002 as compared to that of UCI-1001 to use concentrations with equivalent cellular response.

Supplementary Tables

Table S1. UCI-1001 Analogs IDs

Compound Name	ChemBridge ID	Compound Name	ChemBridge ID
UCI-1002	5377539	UCI-1034	5736021
UCI-1003	5851455	UCI-1035	6095904
UCI-1004	5155545	UCI-1036	5966505
UCI-1005	5740628	UCI-1037	5378562
UCI-1006	5967883	UCI-1038	6190330
UCI-1007	5155500	UCI-1039	7033053
UCI-1008	5483251	UCI-1040	5647127
UCI-1009	5980351	UCI-1041	7093707
UCI-1010	5377259	UCI-1042	6909686
UCI-1011	5973869	UCI-1043	6517329
UCI-1012	5733619	UCI-1044	6638601
UCI-1013	6035637	UCI-1045	5976520
UCI-1014	5852728	UCI-1046	6188254
UCI-1015	5377942	UCI-1047	6642029
UCI-1016	5528399	UCI-1048	7027285
UCI-1017	5626469	UCI-1049	6194121
UCI-1018	5809865	UCI-1050	5786256
UCI-1019	5155650	UCI-1051	5789293
UCI-1020	6389270	UCI-1052	6347428
UCI-1021	5377687	UCI-1054	1799448388
UCI-1022	5988102	**Compound Name**	**MCULE-ID**
UCI-1023	5155560	UCI-1055	2089743290
UCI-1024	6568425	UCI-1056	5854795168
UCI-1025	5378117	UCI-1057	6915753653
UCI-1026	5982724	UCI-1058	7241308976
UCI-1027	5983864	UCI-1059	6474197888
UCI-1028	6333614	UCI-1060	8661269907
UCI-1029	5974846	UCI-1061	5418876942
UCI-1030	5625977		
UCI-1031	5900929		
UCI-1032	6341999		
UCI-1033	6220107		

Table S2. Chemical Structures and ID for screening compounds

Compound#	Structure	ChemBridge ID
Compound#1	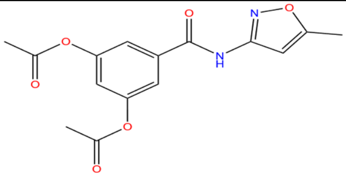<br>5-((5-methylisoxazol-3-yl)carbamoyl)-1,3-phenylene diacetate	7953508
Compound#2	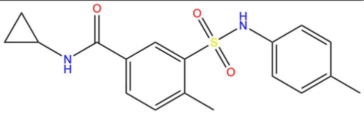<br>*N*-cyclopropyl-4-methyl-3-(*N*-(*p*-tolyl)sulfamoyl)benzamide	7996388
Compound#3	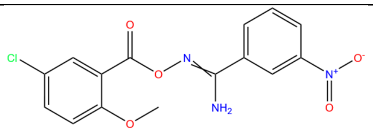<br>*N'*-((5-chloro-2-methoxybenzoyl)oxy)-3-nitrobenzimidamide	7692757
Compound#4	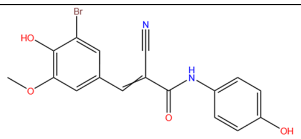<br>3-(3-bromo-4-hydroxy-5-methoxyphenyl)-2-cyano-*N*-(4-hydroxyphenyl)acrylamide	5729375
Compound#5	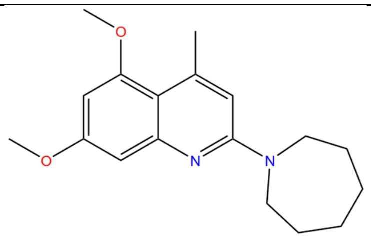<br>2-(azepan-1-yl)-5,7-dimethoxy-4-methylquinoline	5549193
Compound#6	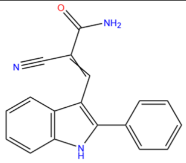<br>2-cyano-3-(2-phenyl-1H-indol-3-yl)acrylamide	5705596
Compound#7	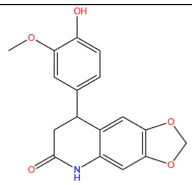<br>8-(4-hydroxy-3-methoxyphenyl)-7,8-dihydro-[1,3]dioxolo[4,5-*g*]quinolin-6(5*H*)-one	9112276

Compound#8	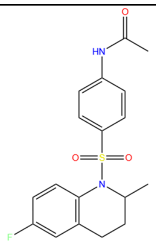 N-{4-[(6-fluoro-2-methyl-3,4-dihydro-1(2H)-quinolinyl)sulfonyl]phenyl}acetamide	7228460
Compound#9	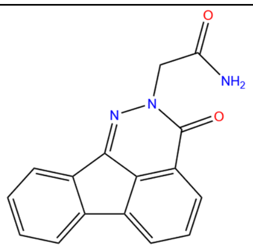 2-(3-oxoindeno[1,2,3-de]phthalazin-2(3H)-yl)acetamide	7145035
Compound#10 UCI-1001	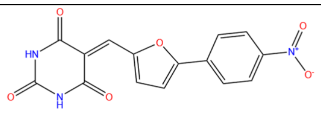 5-((5-(4-nitrophenyl)furan-2-yl)methylene)pyrimidine-2,4,6(1H,3H,5H)-trione	5324771
Compound#11	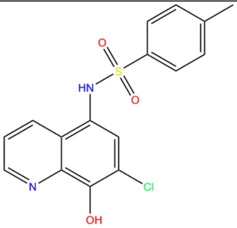 N-(7-chloro-8-hydroxy-5-quinolinyl)-4-methylbenzenesulfonamide	7985231
Compound#12	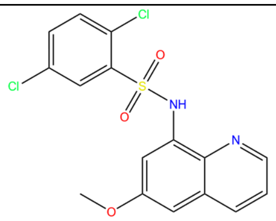 2,5-dichloro-N-(6-methoxy-8-quinolinyl)benzenesulfonamide	5663715
Compound#13	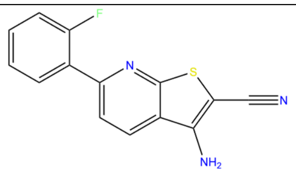 3-amino-6-(2-fluorophenyl)thieno[2,3-b]pyridine-2-carbonitrile	9248417
Compound#14	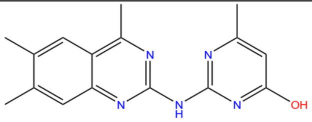 6-methyl-2-[(4,6,7-trimethyl-2-quinazolinyl)amino]-4-pyrimidinol	5803953

Disclaimer/Publisher's Note: The statements, opinions and data contained in all publications are solely those of the individual author(s) and contributor(s) and not of MDPI and/or the editor(s). MDPI and/or the editor(s) disclaim responsibility for any injury to people or property resulting from any ideas, methods, instructions or products referred to in the content.
